# Effects of Phospholipase Dε Overexpression on Soybean Response to Nitrogen and Nodulation

**DOI:** 10.3389/fpls.2022.852923

**Published:** 2022-05-06

**Authors:** Shuaibing Yao, Geliang Wang, Xuemin Wang

**Affiliations:** ^1^Department of Biology, University of Missouri-St. Louis, St. Louis, MO, United States; ^2^Donald Danforth Plant Science Center, St. Louis, MO, United States

**Keywords:** lipid metabolism, nitrogen response, phospholipase D, soybean, nodulation

## Abstract

Nitrogen is a key macronutrient to plant growth. We found previously that increased expression of phospholipase Dε (PLDε), which hydrolyzes phospholipids into phosphatidic acid (PA), enhanced plant growth under nitrogen deficiency in Brassicaceae species Arabidopsis and canola. The present study investigated the effect of *AtPLDε*-overexpression (OE) on soybean (*Glycine max*), a species capable of symbiotic nitrogen fixation. *AtPLDε*-OE soybean plants displayed increased root length and leaf size, and the effect of *AtPLDε*-ΟΕ on leaf size was greater under nitrogen-deficient than -sufficient condition. Under nitrogen deficiency, *AtPLDε*-OE soybean plants had a higher chlorophyll content and activity of nitrogen assimilation-related enzymes than wild-type soybean plants. *AtPLDε*-OE led to a higher level of specific PA species in roots after rhizobium inoculation than wild type. *AtPLDε*-OE soybean plants also increased seed production under nitrogen deprivation with and without nodulation and decreased seed germination in response to high humidity storage and artificial aging. These results suggest that PLDε promotes nitrogen response and affects adversely seed viability during storage.

## Introduction

Soybean is the major source of plant-based protein for human consumption and animal industries and a major crop for oil production (Graham and Vance, 2003). The global production of soybean has increased by 13-fold from 1961 to 2017, but the increase in soybean production is mainly resulted from the increasing planting area of soybean (Liu et al., 2020). To meet the increasing demands for food and energy, increasing soybean yield continues to be the major focus for soybean breeding. Nitrogen (N) for soybean production is supplied from nitrogen fixation and fertilization (Bassi et al., 2018). Soybean can fix nitrogen with the symbiotic help of rhizobia bacteria, but the amount of N from N_2_ fixation is not sufficient to meet the demand for soybean seed production in the field (Salvagiotti et al., 2008). In addition, the biological N fixation has a metabolic cost, which could potentially reduce seed oil and protein contents in seeds. N fertilization has not only greatly increased the crop yields, but it also causes the acid soil and environment pollution (Graham and Vance, 2003). Moreover, the increase in N fertilization in soil is correlated with the decrease in N fixation rate (Tamagno et al., 2018). Thus, breeding the cultivars with high seed yield and N use efficiency is important for soybean breeding.

Phospholipase D (PLD), which hydrolyzes phospholipids, such as phosphatidylcholine (PC) and phosphatidylethanolamine (PE), to produce phosphatidic acid (PA), is involved in plant response to nutrient deprivation, including N and phosphate (Cruz-Ramírez et al., 2006; Li et al., 2006; Hong et al., 2009; Lu et al., 2016). Plants have multiple PLDs and many of them have distinguishable biochemical, regulatory, and physiological functions (Hong et al., 2016). Particularly, overexpression (OE) of *PLDε* enhanced growth under N deficiency in Arabidopsis (Hong et al., 2009) and canola (Lu et al., 2016). Our recent results indicate that one mechanism by which *PLDε* enhances Arabidopsis growth is *via* its interaction with and promotion of autophagy to promote N recycling (Yao et al., 2022). Thus, *PLDε* could be one target for enhancing plant growth under limited N conditions. However, it remains to be determined whether the result from the Brassicaceae plants is applicable to other plant species. In particular, soybean is capable of biological N fixation *via* symbiosis, and how *PLDε*-OE affects soybean growth and rhizobial nodulation is another intriguing question. Therefore, this study was undertaken to investigate the effect of *PLDε*-OE on soybean growth and seed properties in response to N availability and rhizobial nodulation.

## Materials and Methods

### Plant Materials and Growth Conditions

To generate *AtPLDε*-OE soybean plants, the full-length coding region of Arabidopsis *PLDε* (At1g55180) was fused with a HA tag at the 3′ end, and then cloned into a binary vector pZY101 under the control of the CaMV 35S promoter. The resulting vectors were transformed into the *Agrobacterium* strain EHA101 by a freeze–thaw method. Soybean (cv. Williams 82) was subjected to *Agrobacterium*-mediated cotyledonary node transformation (Zeng et al., 2004). Transgenic soybean lines were screened by the resistance to the herbicide Basta and verified by PCR using Arabidopsis *PLDε* gene-specific primers (Supplementary Table 1). Soybeans were grown in a growth chamber at 16 h light 25°C/8 h dark 22°C.

For N treatments, soybean seeds were germinated on vermiculite watered with Hoagland solution containing 6 mM N (KNO_3_) for 7 days in a growth chamber at 16 h light 25°C/8 h dark 22°C, and then transferred to Hoagland solution supplied with 6 mM N. After 7 days, the soybean plants were transferred to Hoagland solution supplied with 6 or 0 mM N for 7 days. For nodulation, soybean seeds were germinated on vermiculite watered with Hoagland solution containing 0.6 mM N (KNO_3_) in a growth chamber at 16 h light 25°C/8 h dark 22°C. After 21 days, the soybean plants were infected with *Bradyrhizobium diazoefficiens* USDA110 (OD_600_ = 0.2) grown in Yeast Mannitol (YM) medium. After infection, plants were maintained under the same condition, and watered with Hoagland solution containing 0.6 mM N until harvest stage.

### RNA Extraction and RT-qPCR

Total RNA was isolated from soybean leaves using the RNeasy Plant Mini Kit (Qiagen), and the cDNAs were synthesized using the qScript cDNA Synthesis Kit (Quanta BioSciences) according to the manufacturer’s manual. Semi-quantitative PCR and real-time qPCR were performed to analysis gene expression. Actin (Glyma18g52780) was used as internal control to normalize the gene expression. The gene-specific primers are listed in Supplementary Table 1.

### Immunoblotting

Total proteins were extracted from the leaves of soybean plants using an extraction buffer (50 mM Tris–HCl, pH 8.0, 150 mM NaCl, 10 mM EDTA, 1 mM PMSF, and 10 mM iodoacetamide). After centrifugation at 2,000 *g* for 5 min at 4°C, the supernatant was subjected to 8% SDS-PAGE and transferred onto a polyvinylidene difluoride membrane. PLDε-HA on the membrane was immunodetected using an anti-HA antibody (Invitrogen 26183; 1:4,000 dilution) as the primary antibody and the HRP-conjugated anti-rabbit antibody (Sigma) as the secondary antibody.

### Assaying Nitrate Reductase, Nitrite Reductase, and Glutamine Synthetase Activities

Soybean leaves were ground in liquid nitrogen and added to an extraction buffer (50 mM Tris–HCl, pH 8.0, 150 mM NaCl, 10 mM EDTA, 10 mM EGTA, 1 mM PMSF, 10 mM iodoacetamide, 0.5% Triton X-100). The extract was centrifuged at 5,000 *g* for 10 min at 4°C and the supernatant was used for enzyme assays. The total protein concentration was measured using Braford assay (Bio-Rad). All the enzyme activities were measured spectrophotometrically. The nitrate reductase activity was measured according to Hageman and Hucklesby (1971) with modifications. Briefly, 20 μl of extracts was added to 380 μl of a reaction buffer (50 mM KH_2_PO_4_, pH 7.5, 20 mM KNO_3_, 0.35 mM NADH) and incubated at 30°C for 30 min. The reaction was stopped by adding 300 μl of 1% (w/v) sulfanilamide in 3 M HCl and 300 μl of 0.02% (w/v) N-1-naphthylethylenediamine dihydrochloride. The absorbance was measured at 540 nm, and NaNO_2_ was used to prepare a standard curve. The nitrite reductase activity was measured according to Joy and Hageman (1966). Briefly, 25 μl of extracts were added to a 500 μl reaction buffer (50 mM KH_2_PO_4_, pH 7.5, 1 mM NaNO_2_, 1 mM methyl viologen), and the reaction was started by adding 150 μl of 57.4 mM Na_2_S_2_O_4_ in 290 mM NaHCO_3_. After incubated at 30°C for 60 min, 40 μl of the reaction mixture was added to 360 μl H_2_O in a new tube, followed by adding 300 μl of 1% (w/v) sulfanilamide in 3 M HCl and 300 μl of 0.02% (w/v) N-1-naphthylethylenediamine dihydrochloride. A_540_ of the reaction mixture was measured, and NaNO_2_ was used to generate standard curve. The glutamine synthetase activity was measured according to Liu et al. (2010). Briefly, 50 μl of extracts was added to 100 μl of a reaction buffer (70 mM MOPS, pH 6.8, 100 mM L-glutamic acid, 50 mM MgSO_4_, 15 mM NH_2_OH, 15 mM ATP) at 37°C for 30 min. The reaction was stopped by addition of 450 μl of 88 mM FeCl_3_, 670 mM HCl, and 200 mM trichloroacetic acid. The absorbance was measured at 498 nm, and blank controls were obtained by incubating protein extracts in the reaction buffer without addition of ATP. GHA (γ-glutamyl hydroxymate) was used as standard.

### Lipid Extraction and Profiling

Total lipids were extracted and analyzed by electrospray ionization–tandem mass spectrometry (ESI–MS/MS) according to Welti et al. (2002). Briefly, the samples were immediately immersed into 3 ml of preheated isopropanol containing 0.01% butylated hydroxytoluene (BHT) at 75°C for 15 min and then added with 1.5 ml of chloroform and 0.6 ml of water. After incubated in a shaking incubator at room temperature for 1 h, the extracts were transferred to glass tubes. The samples were then extracted with chloroform/methanol (2:1) containing 0.01% BHT four times. The combined extracts were washed once with 1 M KCl and then water. The lower phase was dried under nitrogen gas and resuspended with chloroform (250 μl chloroform/mg dry weight). Lipids were introduced by direct infusion to mass spectrometer (API4000, Sciex), and scanned in the positive ion mode as described previously (Welti et al., 2002). The seed oil contents were analyzed using gas chromatography (GC) according to Guo et al. (2014).

### Seed Aging and Germination Assay

For artificial aging, soybean seeds were placed in a sealed container with a beaker filled with water and then stored in an incubator at 45°C for 5 days. The fresh harvested, stored under high humidity condition, and artificially aged seeds were surface sterilized with chloride gas and then washed with sterilized water five times. After placed on sterilized wet filter paper in petri dish, seeds were germinated at 25°C.

### Chlorophyll Content

Approximately 20 mg of leaves were incubated in 2 ml of 95% ethanol and then placed in a rotating incubator overnight or until the leaf is bleached at 4°C under the dark. The absorbance was measured at 664 nm and 649 nm. The contents of chlorophyll a and b were calculated as C_a + b_ = 5.24A_664_ + 22.24A_649_ and normalized by fresh weight.

## Results

### Overexpression of *AtPLDε* Promoted Soybean Growth and Nitrate Assimilation

**Figure 1:**
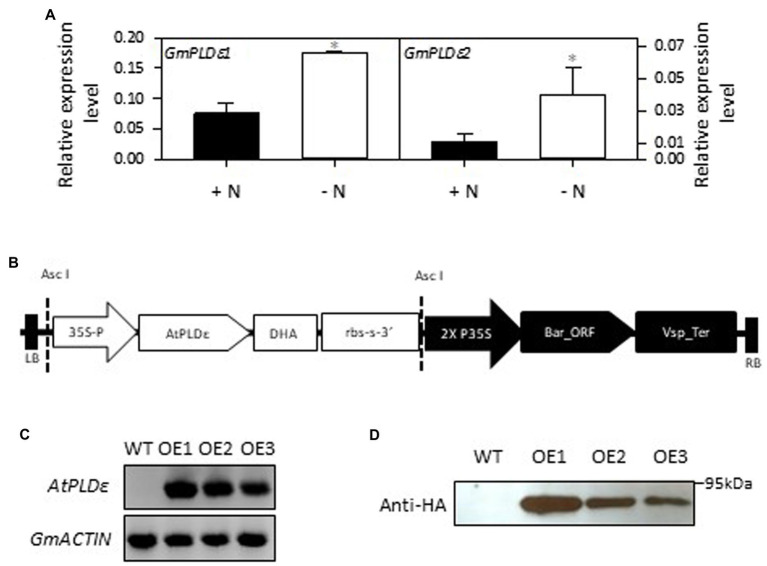
Generation and verification of *AtPLDε*-overexpressing soybean plants. **(A)** RT-qPCR analysis of relative expression level of *GmPLDε1* and *GmPLDε2* in the leaves of WT under nitrogen-sufficient and -deficient conditions. Two-week-old soybeans grown on nitrogen rich liquid medium were transferred to nitrogen rich (6 mM N) or free (0 mM) liquid medium for 7 days. The values are means ± SD (*n*=3). One asterisk indicates statistical differences compared with nitrogen sufficient conditions determined by student’s *t* test (*p* < 0.05). **(B)** Schematic diagram of the binary vectors used to overexpress *AtPLDε* in soybean. Full-length coding region of *AtPLDε* was fused with a HA tag at the 3′ end, and then cloned into a binary vector PZY101 under the control of the CaMV 35S promoter. The Basta resistance gene was used as the selectable marker. The T-DNA left border (LB) and right border (RB) are shown. **(C)** Expression of *AtPLDε* in wildtype (cultivar Williams 82) and three independent transgenic soybean lines were determined by semi-quantitative RT-PCR. **(D)** Immunoblotting detection of hemagglutinin (HA)-tagged AtPLDε in WT and *AtPLDε* OE soybean plants.

The soybean genome contains two *PLDεs*, *GmPLDε1* (Glyma07g01310) and *GmPLDε2* (Glyma15g02710), which are homologous to Arabidopsis *PLDε* (Zhao et al., 2012). The amino acid sequence identity between AtPLDε and GmPLDε1/GmPLDε2 is 62% and 62.2%, respectively (Supplementary Figure 1A). To examine how soybean *PLDε* responds to N deficiency, we measured the transcript level of two *PLDεs* in soybean under N-sufficient and -deficient conditions. When 2-week-old soybean seedlings were transferred to nitrogen-rich or -free medium for 7 days, the transcript level of *GmPLDε1* and *GmPLDε2* in soybean leaves was increased under the N-deficient condition by 132% and 254%, respectively (Figure 1A).

To study the effect of PLDε on soybean growth, we overexpressed *PLDε* from Arabidopsis in soybean (cultivar William 82) under the CaMV-35S promoter (Figure 1B), based on the previous reports that Arabidopsis PLDε enhanced plant growth in Arabidopsis and canola under N deprivation (Hong et al., 2009; Lu et al., 2016). The expression of *AtPLDε* in three independent transgenic lines was verified by semi-quantitative PCR and the production of hemagglutinin (HA) tagged-PLDε was confirmed by immunoblotting with an anti-HA antibody (Figures 1C,D). *AtPLDε*-OE1 showed the highest expression level of *PLDε* among three lines. T3 generation of homozygous transgenic plants was used for the future analysis. In contrast with WT soybean, the transcript level of *GmPLDε1* and *GmPLDε2* in *AtPLDε*-OE1was not induced under N-deficient condition (Supplementary Figure 1B).

**Figure 2:**
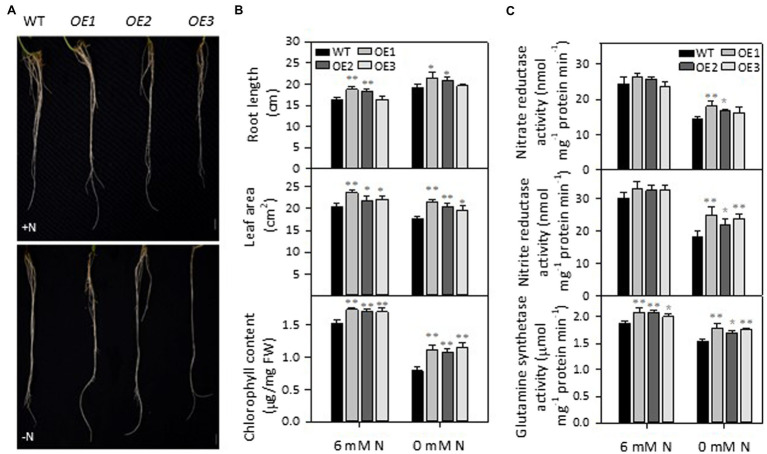
Phenotype of *AtPLDε*-overexpressing soybean plants. **(A)** Root morphology of WT and *AtPLDε* OE soybean plants under nitrogen-sufficient and -deficient conditions. Two-week-old soybeans grown on nitrogen rich liquid medium were transferred to nitrogen rich (6 mM N) or free (0 mM) liquid medium for 7 days. Scale bar = 1 cm. **(B)** Root length, leaf area, and chlorophyll content of WT and *AtPLDε* OE soybean plants under nitrogen-sufficient and -deficient conditions. The values are means ± SD (*n* = 3, *r* = 5). *Denotes statistical significance at *p* < 0.05 and **denotes statistical significance at *p* < 0.001 compared with WT as determined by one-way ANOVA (Duncan’s multiple range test). **(C)** Activity of nitrate reductase, nitrite reductase, and glutamine synthetase (normalized to equal amounts of protein) in the leaves of WT, and *AtPLDε* OE soybean. The values are means ± SD (*n* = 3, *r* = 5). *Denotes statistical significance at *p* < 0.05 and **denotes statistical significance at *p* < 0.001 compared with WT as determined by one-way ANOVA (Duncan’s multiple range test).

To test the effect of *PLDε*-OE on soybean response to N deprivation, 2-week-old soybean seedlings grown in Hoagland solution containing 6 mM N were transferred to Hoagland solution supplied with 6 or 0 mM N for 7 days. Compared with wild-type (WT), *AtPLDε*-OE soybean plants displayed larger leaf area, and higher chlorophyll content under both N-sufficient and -deficient conditions (Figures 2A,B). However, the difference between *AtPLDε*-OE and WT plants in leaf area and chlorophyll content was greater under the N-deficient than N-sufficient condition. The chlorophyll content of three *AtPLDε*-OE lines was, on average, 41% higher than that of WT under N deficiency whereas that difference was 12% under sufficient N. The leaf area of three *AtPLDε*-OE lines was, on average, 16% higher than that of WT under N deficiency whereas that difference was 10% under sufficient N. Under N deficiency, leaf growth was inhibited, and chlorophyll content was decreased, but the primary root elongation was promoted. The primary root length of *AtPLDε*-OE1 and -OE2 with higher expression of *AtPLDε* was, on average, 14% longer under N-sufficient but 10% longer than that of WT under N-deficient condition (Figure 2B). These results indicate that *AtPLDε*-OE plants display less N-deficiency-induced growth alterations than WT, and *PLDε*-OE enhances leaf growth and chlorophyl content more under N-deficient than N-sufficient condition (Supplementary Figure 2).

Nitrate is the major form of N in soil taken up by plants, where it is reduced sequentially by nitrate reductase (NR) and nitrite reductase (NiR) to ammonium that is incorporated into amino acids by glutamine synthetase (GS; Masclaux-Daubresse et al., 2010). The activities of NiR, and GS in *AtPLDε*-OE leaves were higher than those of WT under sufficient N, and the effect was greater under N deficiency (Figure 2C). Nitrate deficiency led to a decrease in NR, NiR and GS in both WT and *AtPLDε*-OE leaves. However, compared to those grown under sufficient N, *AtPLDε*-OE soybean still maintained relatively higher activities of NR, NiR and GS when grown under N deficiency (Supplementary Figure 3). Three *AtPLDε*-OE lines had an average of 13% higher relative NR activity and 18% higher relative NiR activity than those of WT plant. The GS activity in *AtPLDε*-OE1 and -OE3 leaves was 7% higher than that of WT (Supplementary Figure 3). These results suggest that *PLDε*-OE enhances N assimilation.

### *PLDε*-OE Enhanced Rhizobium-Promoted Growth Without Apparent Effect on Nodulation

**Figure 3:**
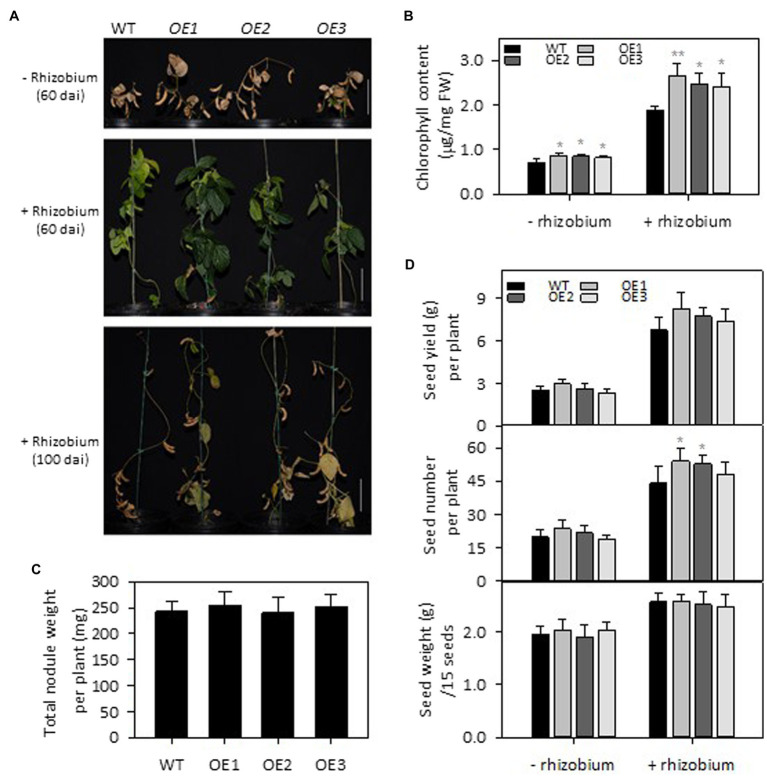
Effects of *PLDε* alterations on seed yield under nitrogen deficiency with or without nodulation. **(A)** Morphology of WT and *AtPLDε* OE soybean plants under nitrogen-deficient conditions 60 or 100 days after infection (dai) with *Bradyrhizobium diazoefficiens* USDA110. Three-week-old soybeans grown on vermiculite supplied with 0.6 mM N were infected with *Bradyrhizobium diazoefficiens* USDA110. Scale bar = 10 cm. **(B)** Chlorophyll content of leaves from WT and *AtPLDε* OE soybean plants under nitrogen-deficient conditions 28 days after infection. The values are means ± SD of five biological replicates. *Denotes statistical significance at *p* < 0.05 and **denotes statistical significance at *p* < 0.001 compared with WT as determined by one-way ANOVA (Duncan’s multiple range test). **(C)** Total nodule weight collected at 28 days after infection (*n* = 5). **(D)** Seed yield: total seed number: and 15-seed weight (*n* = 6). *Denotes statistical significance at *p* < 0.05 compared with WT as determined by one-way ANOVA (Duncan’s multiple range test).

To test the effect of *PLDε*-OE on soybean growth under N deficiency with and without nodulation, 3-week-old soybean seedlings grown on vermiculite watered with Hoagland solution containing 0.6 mM N were infected with *B. diazoefficiens* USDA110. The inoculation of soybean plants with rhizobia enhanced plant growth under N deprivation, but the enhancement was greater in *AtPLDε*-OE than WT plants (Figures 3A,B). Four weeks after infection, the leaf chlorophyll content in three *AtPLDε*-OE lines with inoculation was, on average, 33% higher than that of WT, whereas that in three *AtPLDε*-OE lines at the same growth stage without inoculation was 19% higher than that of WT (Figure 3B). However, there was no significant difference among the total weight of nodules collected from WT and *AtPLDε*-overexpressing soybean roots 28 days after inoculation (Figure 3C).

Compared with WT, the seed yield in *PLDε*-OE1 and -OE2 without inoculation was increased by an average of 12%, and the seed yield in *PLDε*-OE1 and -OE2 with inoculation was increased by an average of 18% (Figure 3D). The seed number was significantly increased in *PLDε*-OE1 and -OE2 plants with inoculation, which had higher expression of *AtPLDε*, and the average seed weight of WT and *AtPLDε*-OE soybean lines remained similar (Figure 3D).

### *PLDε*-OE Changed Lipid Composition in Soybean

**Figure 4:**
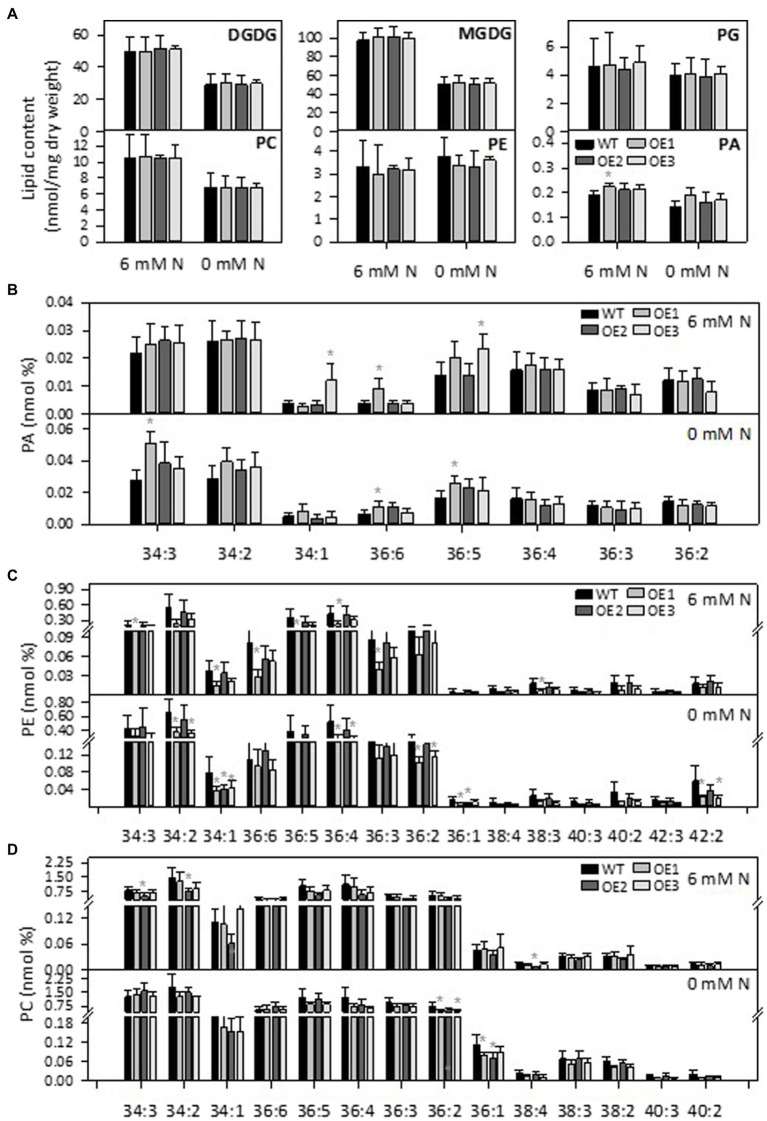
Effect of *PLDε* alterations on lipid content and composition. **(A)** Lipid contents in leaves of WT and *AtPLDε* OE soybean. Two-week-old soybeans grown on nitrogen rich liquid medium were transferred to nitrogen rich or free liquid medium for 7 days. The values are means ± SD of five biological replicates. *Denotes statistical significance at *p* < 0.05 compared with WT as determined by one-way ANOVA (Duncan’s multiple range test). PA **(B)**, PE **(C)**, and PC **(D)** molecular species in leaves of WT and *AtPLDε* OE soybean.

PLD*ε* hydrolyzes phospholipids to produce PA. To analyze the effect of *PLDε*-OE on lipids composition, we used an ESI-MS/MS approach to quantitatively profile lipid species in leaves from Hoagland solution-grown plants with and without N. After 7 days of N starvation, the amounts of galactolipids monogalactosyldiacylglycerol (MGDG) and digalactosyldiacylglycerol (DGDG), which are the major lipids of chloroplast membranes, and PC were greatly decreased in WT and *AtPLDε*-OE soybean plants (Figure 4A). The total PA level tended to be higher in *PLDε*-OE lines, but only *PLDε*-OE1 that had the highest expression of *AtPLDε* displayed a significantly higher level of total PA than WT under sufficient N (Figure 4A). However, when PA molecular species were compared, *AtPLDε*-OE1 leaves exhibited increases over WT in some PA species, including 36:6 PA under N-sufficient and -deficient conditions, and 34:3 PA and 36:5 PA under N deficiency (Figure 4B). *PLDε*-OE3 leaves showed an increase in 36:5 PA, but *PLDε*-OE2 leaves had no significant PA increase (Figure 4B). On the other hand, most PE and PC species, including 34:2, 34:1, 36:4, 36:2 and 36:1 were decreased in *AtPLDε*-OE plants under N deficiency (Figures 4C,D).

**Figure 5:**
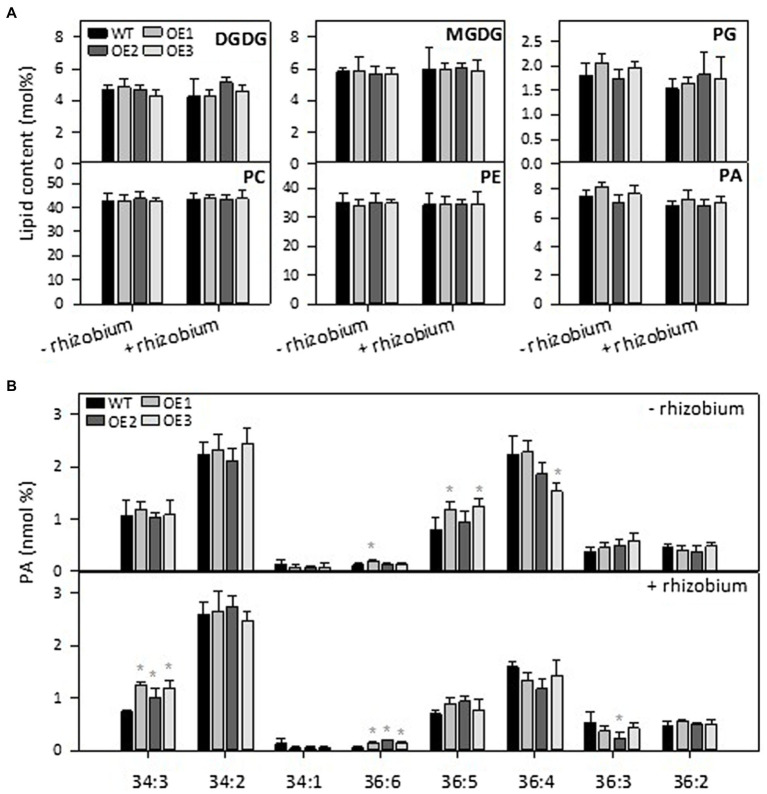
Effect of *PLDε* alterations on lipid content under nitrogen deficiency with or without nodulation. **(A)** Lipid contents in roots of WT and *AtPLDε* OE soybean plants under nitrogen-deficient conditions. Three-week-old soybeans grown on vermiculite supplied with 0.6 mM N were infected with *Bradyrhizobium diazoefficiens* USDA110 and maintained for 28 days. The values are means ± SD of four biological replicates. *Denotes statistical significance at *p* < 0.05 compared with WT as determined by one-way ANOVA (Duncan’s multiple range test). **(B)** PA molecular species in roots of WT and *AtPLDε* OE soybean.

We then analyzed lipid species in roots and nodules of *AtPLDε*-OE and WT plants grown in vermiculites with and without nodulation, Total lipids were extracted from nodules and roots with and without inoculation 28 days after rhizobium inoculation and analyzed by ESI-MS/MS. There were no significant differences on lipid contents (mole percentage) of MGDG, DGDG, phosphatidylglycerol (PG), PC, and PE in roots or nodules between WT and *PLDε*-OE soybean (Figure 5A; Supplementary Figure 4). Compared to roots, PC contents were higher in nodules and PE contents were lower in nodules. Without inoculation, 36:5 PA was higher in roots of *AtPLDε*-OE1 and OE3 lines, but 36:4 PA in OE3 was lower than WT roots, whereas 34:3 PA and 36:6 PA in roots was higher in all three *AtPLDε*-OE lines than WT after rhizobium nodulation (Figure 5B).

### *PLDε*-OE Increased Seed Production and Changed Fatty Acid Composition of Seed Oil

**Figure 6:**
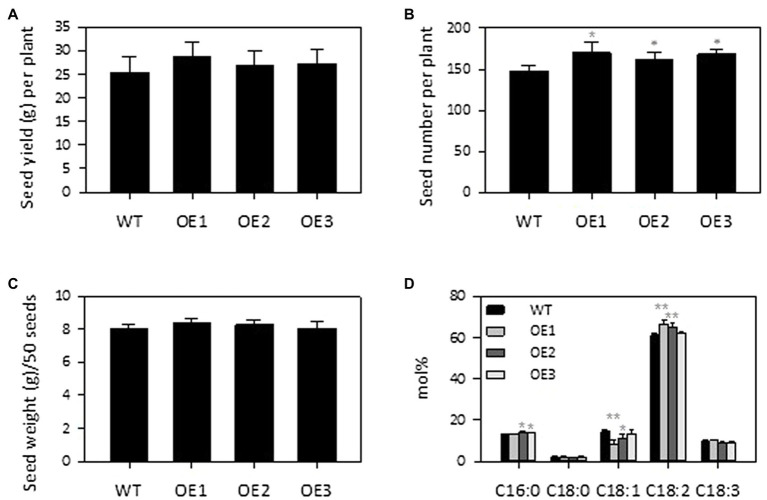
Effect of *PLDε* alterations on seed yield **(A)**, seed number **(B)**, 50-seed weight **(C)** and fatty acid composition of seed oil **(D)**. The seeds were harvested from WT and *AtPLDε* OE soybean grown on normal soils in greenhouse (*n* = 6 plants). The values are means ± SD. *Denotes statistical significance at *p* < 0.05 and **denotes statistical significance at *p* < 0.001 compared with WT as determined by one-way ANOITA (Duncan’s multiple range test).

Soybean is a major protein and oil crop, so we assessed the effect of *PLDε*-OE on seed and oil production of soybean plants grown in soil. *AtPLDε*-OE and WT soybean plants were grown side by side to maturity in greenhouse. The seed number per plant was higher in all three *AtPLDε*-OE lines than WT. The seed number per plant in three *AtPLDε*-OE lines was, on average, 13% higher than that of WT (Figure 6B). There were no significant differences among the total seed yield and seed weight for *AtPLDε*-OE and WT (Figures 6A,C). The plants in soil grew much better than those grown under the vermiculite condition, and the seed yield per plant in soil was about 3-fold and 9-fold higher than that of the vermiculite-grown plants with and without nodulation, respectively (Figures 3D, 6A).

The total seed oil content of *AtPLDε*-OE soybean lines was comparable with that of WT soybean (Supplementary Figure 5), whereas the fatty acid composition exhibited a change in levels of oleic acid (C18:1) and linoleic acid (C18:2) in *AtPLDε*-OE soybean seeds (Figure 6D). Two of the *PLDε*-OE lines which had higher expression of *AtPLDε* displayed a significant decrease in the oleic acid content with an increased linolenic acid content; significant changes in the two fatty acids from WT seeds (Figure 6D). In particular, *AtPLDε*-OE1 with the highest expression of *AtPLDε* had 41% decrease in oleic acid and 9% increase in linoleic acid than WT (Figure 6D).

### *PLDε*-OE Decreased Seed Viability After Storage and Artificial Aging

**Figure 7:**
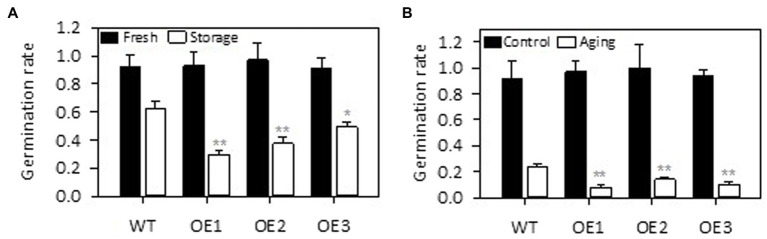
Overexpression of *AtPLDε* decreased seed viability under high humidity storage and artificial aging. The fresh harvested seeds, seeds stored under low temperature (4°C) and high humidity conditions for 12 months **(A)**, and seeds stored under high temperature (45°C) and high humidity conditions for 5 days **(B)** were used for germination rate test (*n* = 4 sets of 25 seeds). The values are means ± SD. *Denotes statistical significance at *p* < 0.05 and **denotes statistical significance at *p* < 0.001 compared with WT as determined by one-way ANOVA (Duncan’s multiple range test).

We further tested the effect of *PLDε*-OE on seed viability because earlier studies indicated that alterations of *PLDα* expression affected seed viability in Arabidopsis (Devaiah et al., 2007) and soybean during storage (Lee et al., 2012; Zhang et al., 2019a). The germination rate of freshly harvested *AtPLDε*-OE and WT soybean seeds was similar (Figure 7A). After stored under a low temperature (4°C) and high humidity condition for 12 months, the germination rate of soybean seed was dramatically decreased in *AtPLDε*-OE seeds (Figure 7A). Compared with WT seeds, the germination rate of *AtPLDε*-OE1, OE2, and OE3 seeds was decreased by 53%, 41%, and 21%, respectively. High temperature and high humidity can decrease the seed viability by accelerating deterioration process (Zhang et al., 2019b). To further verify the role of PLDε in seed storability, we exposed fresh seeds to a high temperature (45°C) and high humidity condition (100%) for 5 days to accelerate seed aging. *AtPLDε*-OE displayed greater sensitivity to this artificial aging than WT seeds. The germination rate of *AtPLDε*-OE1, OE2, and OE3 seeds was on average 50% lower than WT seeds (Figure 7B). These results suggest that *PLDε*-OE reduces seed viability after storage and artificial aging.

## Discussion

The present study showed that *AtPLDε*-OE in soybean displayed higher leaf area, chlorophyll content, and activities of nitrogen-assimilating enzymes than WT plants. In addition, *AtPLDε*-OE soybean exhibited higher seed production than WT. The increases were generally greater under N deficiency than sufficient N. Furthermore, the effect of *PLDε*-OE was tested under different growth conditions, such as soil and vermiculite with and without nodulation. Under all conditions tested, *AtPLDε*-OE1, which displayed the highest level of *PLDε* expression, showed higher seed production than WT. These results indicate that *PLDε* plays a positive role in plant response to nitrogen deprivation, which is consistent with the previous reports that *PLDε*-OE in Arabidopsis and canola enhanced plant growth and seed yield under nitrogen deprivation (Hong et al., 2009; Lu et al., 2016). Thus, the positive effect of *PLDε*-OE in plant growth under N deficiency expands beyond Brassicaceae species to include soybean.

The legume soybean can interact with rhizobia for biological N fixation. A study involving *n*-butanol-suppressed production of PA by PLD inhibited the nodule formation in soybean, suggesting that PLD and its derived PA might be involved in soybean-rhizobium interaction and nodule development (Zhang et al., 2021). Another study indicated that PA was required for cell fusion of *Epichloë festucae* and the symbiotic interaction between *E. festucae* and *Lolium perenne* (Hassing et al., 2020). In addition, the previous studies have shown that PA is one important lipid mediator involved in different cellular processes, including plant and microbial interactions (reviewed in Li and Wang, 2019). Our present study indicates that *AtPLDε*-OE in soybean did not affect nodule formation in terms of total nodule weight. Another report indicated that OE of *PLDα1* in soybean hairy roots suppressed nodule formation (Zhang et al., 2021). The soybean genome has 18 genes encoding PLDs. Based on sequence similarity and domain structure, these PLDs are classified into six types, α(3), β(4), γ, δ(5), ε(2), and ζ(3; Zhao et al., 2012). Different PLDs may differ in substrate preference, subcellular localization, and expression pattern, as those PLDs characterized from Arabidopsis (Hong et al., 2016). These multiplicity and diverse properties of PLD could mean that multiple members of the PLD family may contribute to the modulation of soybean-srhizobium interactions and nodule formation.

Among 12 PLDs in Arabidopsis, only PLDε was found to be involved in N deficiency response (Hong et al., 2009), and its expression increased in response to N deficiency (Yao et al., 2022). In this study, the expression of soybean *PLDε1* and *PLDε2* was induced by nitrogen starvation. In Arabidopsis, nitrogen deficiency induced a change in membrane association of PLDε from association primarily with the plasma membrane under sufficient nitrogen to a substantial presence in intracellular membranes under nitrogen limitation (Yao et al., 2022). In addition, Arabidopsis PLDε was found to interact with ATG8, delipidate ATG8-PE, and promote autophagy in Arabidopsis response to nitrogen deficiency (Yao et al., 2022). In addition, PA interacts with different proteins and affects the enzymatic activity and/or subcellular localization of its binding proteins (Kim and Wang, 2020). Lipid analyses of three *AtPLDε*-OE lines revealed that only *AtPLDε*-OE1 showed a higher level of total PA in leaves than WT, which could result from the level of overexpression as *AtPLDε*-OE1 displayed the highest level of *AtPLDε* expression. However, after rhizobium nodulation, all three lines of *AtPLDε*-OE showed a higher level of 34:3 and 36:6 PA species in roots than WT. Thus, the PLDε’s effect could result from its multifaceted effects, including its enzymatic activity on promoting autophagy and regulatory effects *via* PA to promote growth under nitrogen limitation.

An adverse effect of *PLDε*-OE is decreases in seed viability during storage. High temperature and high humidity during seed development and seed storage can cause loss of seed viability (Zhang et al., 2019b). An early study showed that knockout of *PLDα1* in Arabidopsis enhanced seed resistance to aging during storage and under high temperature and humidity conditions (Devaiah et al., 2007). In addition, knockdown (KD) of *PLDα* in soybean increased seed germination in response to natural aging (Lee et al., 2012). After natural aging, the oleic acid level was decreased, and the linoleic acid was increased in both WT and *PLDα*-KD seeds. Meanwhile, *PLDα* KD seeds displayed higher oleic acid level and lower linoleic acid level than WT seeds (Lee et al., 2012). A recent study showed that seeds harvested from *PLDα1*-KD soybean plant grown under high temperature and high humidity conditions displayed increased germination rate compared with WT seeds (Zhang et al., 2019a). Our study showed that *AtPLDε*-OE seeds had a lower oleic acid level, and higher linoleic acid level than WT seeds, and displayed reduced germination rate in response to aging. These results suggest that the changes in oleic acid and linoleic acid ratios may be associated with seed viability during aging.

In summary, this study indicates that increased *PLDε* expression promotes soybean growth, and the enhancement is greater in plants grown in N-deficient than -sufficient conditions, which is not affected by rhizobial nodulation. The study tested and verified the PLDε effect beyond Brassicaceae species, indicates a broader applicability, and provides impetus for future mechanistic investigations. Meanwhile, the result suggests that increased *PLDε* expression decreases seed viability during storage. Thus, organ-specific manipulations of *PLDε* would be needed to enhance growth while maintaining high quality, viable seeds.

## Data Availability Statement

The original contributions presented in the study are included in the article/Supplementary Material, and further inquiries can be directed to the corresponding author.

## Author Contributions

SY designed and performed the experiments and wrote the manuscript. GW constructed OE transgene and confirmed transgenic soybean. XW proposed and supervised the study and revised the manuscript. All authors discussed the results and commented on the manuscript. All authors contributed to the article and approved the submitted version.

## Funding

This work was supported by grants from Agriculture and Food Research Initiative (AFRI) award no. 2016-67013-24429/project accession number 1007600 and 2020-67013-30908/project accession number 1022148 from the USDA National Institute of Food and Agriculture.

## Conflict of Interest

The authors declare that the research was conducted in the absence of any commercial or financial relationships that could be construed as a potential conflict of interest.

## Publisher’s Note

All claims expressed in this article are solely those of the authors and do not necessarily represent those of their affiliated organizations, or those of the publisher, the editors and the reviewers. Any product that may be evaluated in this article, or claim that may be made by its manufacturer, is not guaranteed or endorsed by the publisher.
