# Supplementary material for: Effects of Phospholipase Dε Overexpression on Soybean Response to Nitrogen and Nodulation
Source: Front Plant Sci. 2022 May 6;13:852923. doi: 10.3389/fpls.2022.852923 (PMC9121066; doi:10.3389/fpls.2022.852923)
Supplement: Supplementary file 1 [file Presentation_1.PPTX]

## Slide 1
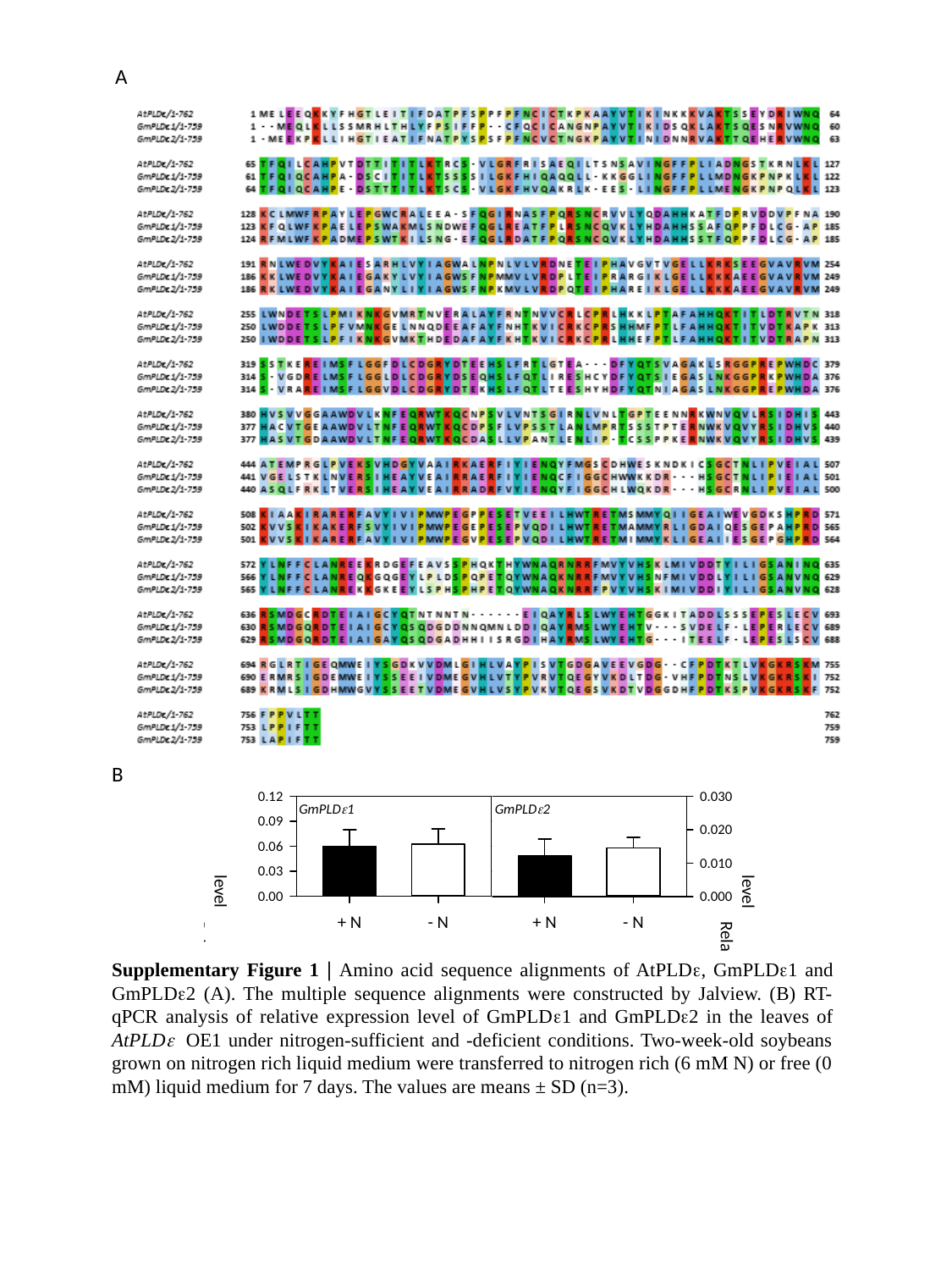

A
B
Supplementary Figure 1 | Amino acid sequence alignments of AtPLDe, GmPLDe1 and GmPLDe2 (A). The multiple sequence alignments were constructed by Jalview. (B) RT-qPCR analysis of relative expression level of GmPLDe1 and GmPLDe2 in the leaves of AtPLDe OE1 under nitrogen-sufficient and -deficient conditions. Two-week-old soybeans grown on nitrogen rich liquid medium were transferred to nitrogen rich (6 mM N) or free (0 mM) liquid medium for 7 days. The values are means ± SD (n=3).

## Slide 2
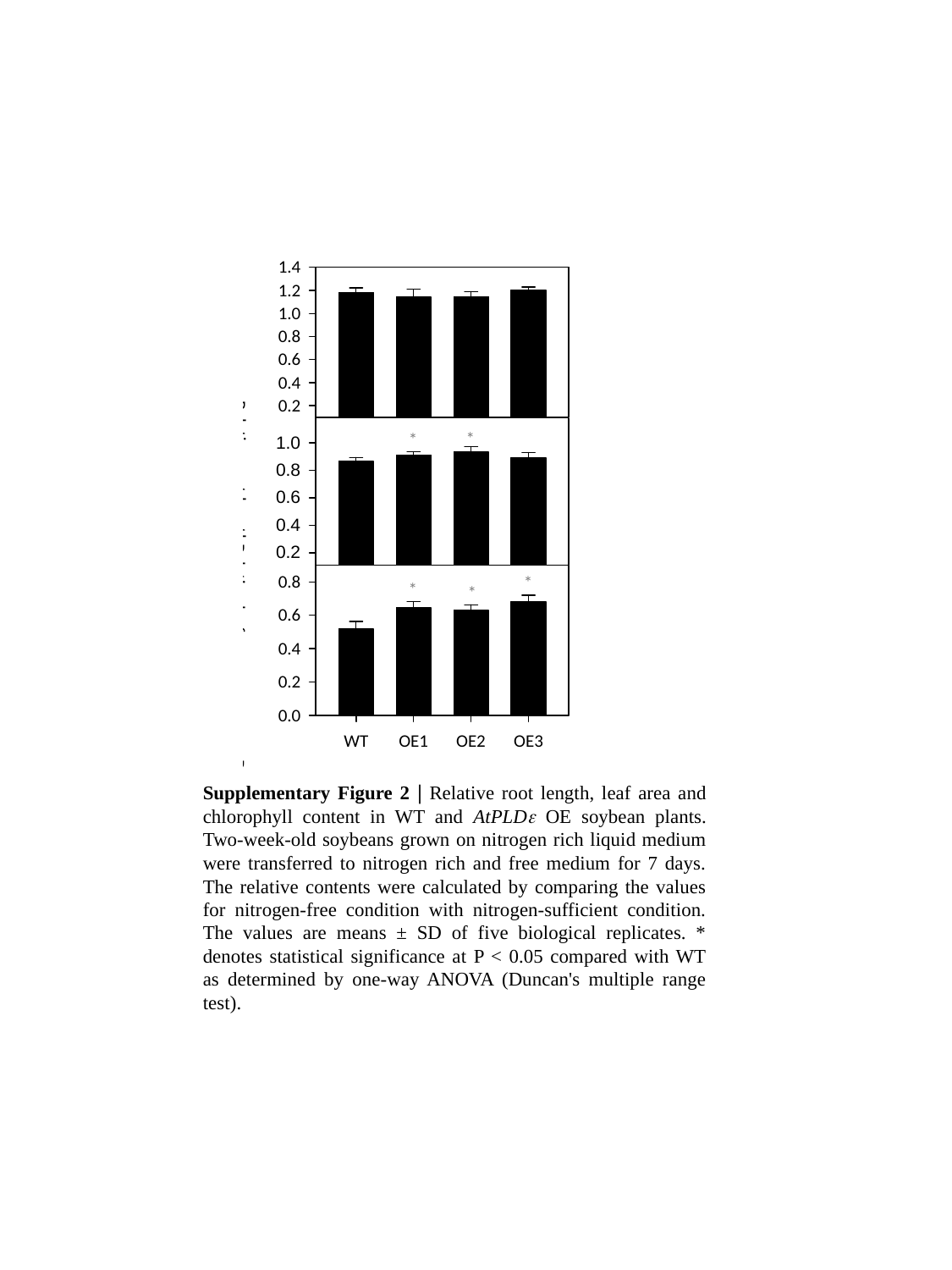

Supplementary Figure 2 | Relative root length, leaf area and chlorophyll content in WT and AtPLDe OE soybean plants. Two-week-old soybeans grown on nitrogen rich liquid medium were transferred to nitrogen rich and free medium for 7 days. The relative contents were calculated by comparing the values for nitrogen-free condition with nitrogen-sufficient condition. The values are means ± SD of five biological replicates. * denotes statistical significance at P < 0.05 compared with WT as determined by one-way ANOVA (Duncan's multiple range test).

## Slide 3
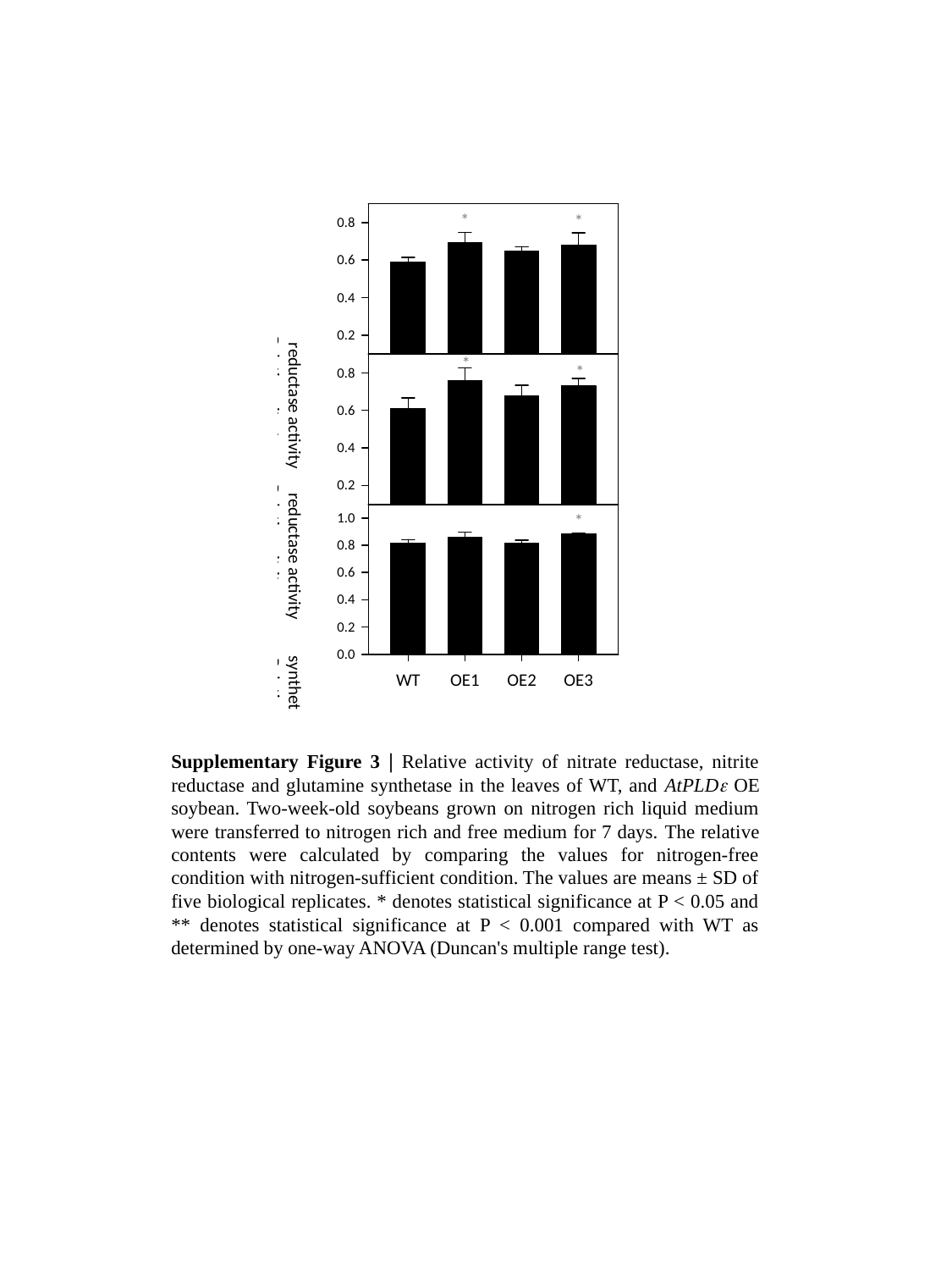

Supplementary Figure 3 | Relative activity of nitrate reductase, nitrite reductase and glutamine synthetase in the leaves of WT, and AtPLDe OE soybean. Two-week-old soybeans grown on nitrogen rich liquid medium were transferred to nitrogen rich and free medium for 7 days. The relative contents were calculated by comparing the values for nitrogen-free condition with nitrogen-sufficient condition. The values are means ± SD of five biological replicates. * denotes statistical significance at P < 0.05 and ** denotes statistical significance at P < 0.001 compared with WT as determined by one-way ANOVA (Duncan's multiple range test).

## Slide 4
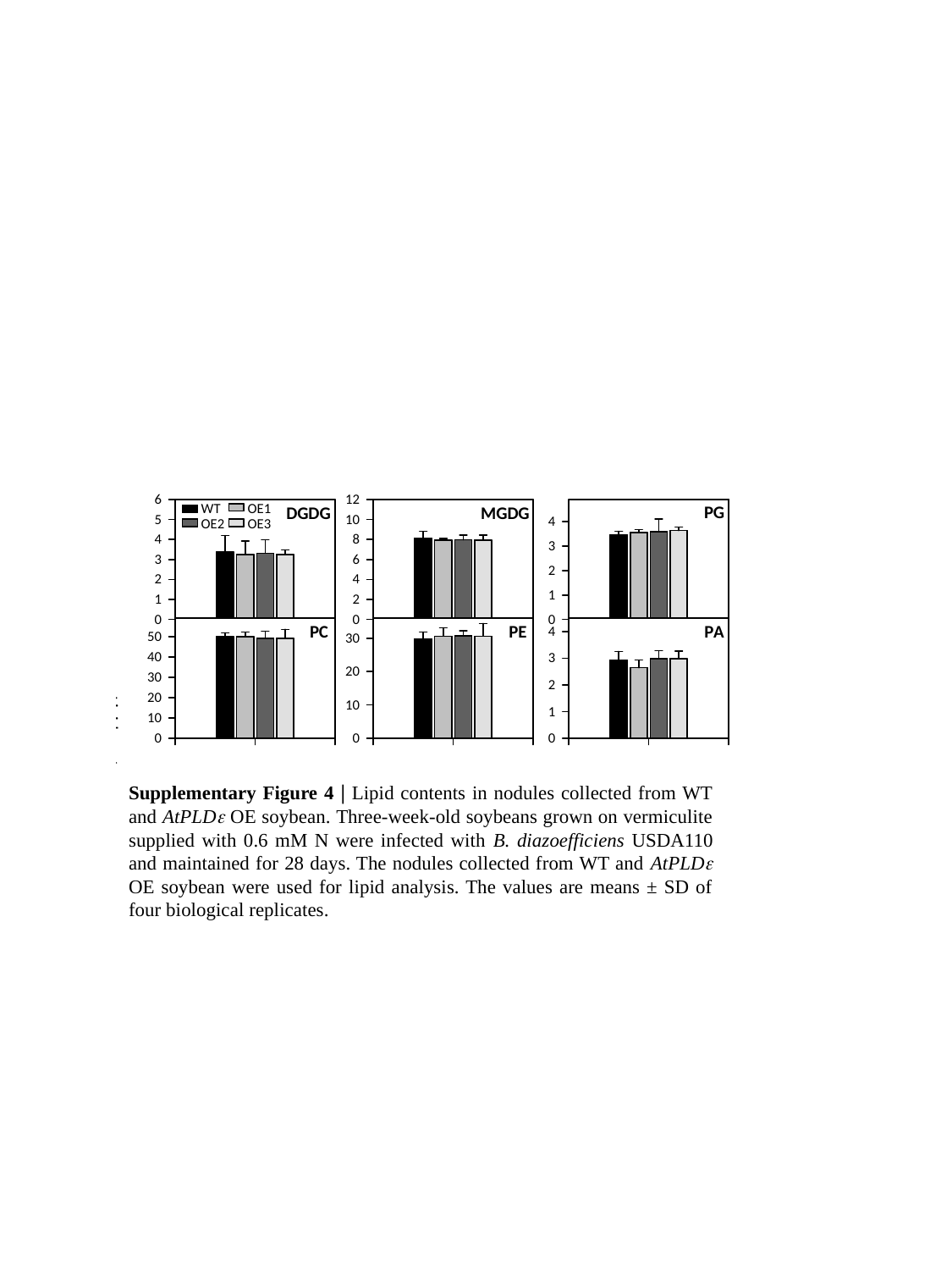

Supplementary Figure 4 | Lipid contents in nodules collected from WT and AtPLDe OE soybean. Three-week-old soybeans grown on vermiculite supplied with 0.6 mM N were infected with B. diazoefficiens USDA110 and maintained for 28 days. The nodules collected from WT and AtPLDe OE soybean were used for lipid analysis. The values are means ± SD of four biological replicates.

## Slide 5
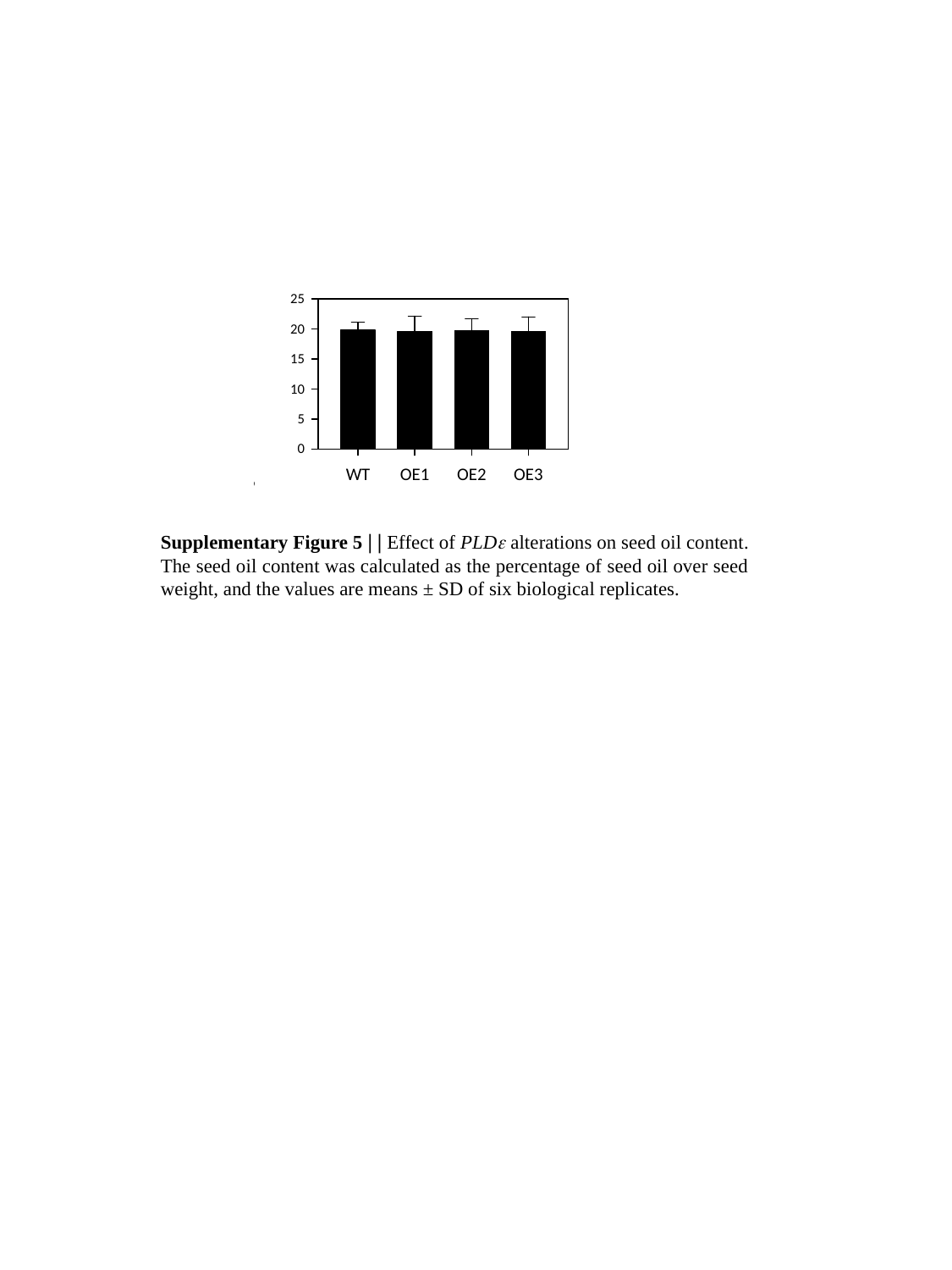

Supplementary Figure 5 | | Effect of PLDe alterations on seed oil content. The seed oil content was calculated as the percentage of seed oil over seed weight, and the values are means ± SD of six biological replicates.

## Slide 6
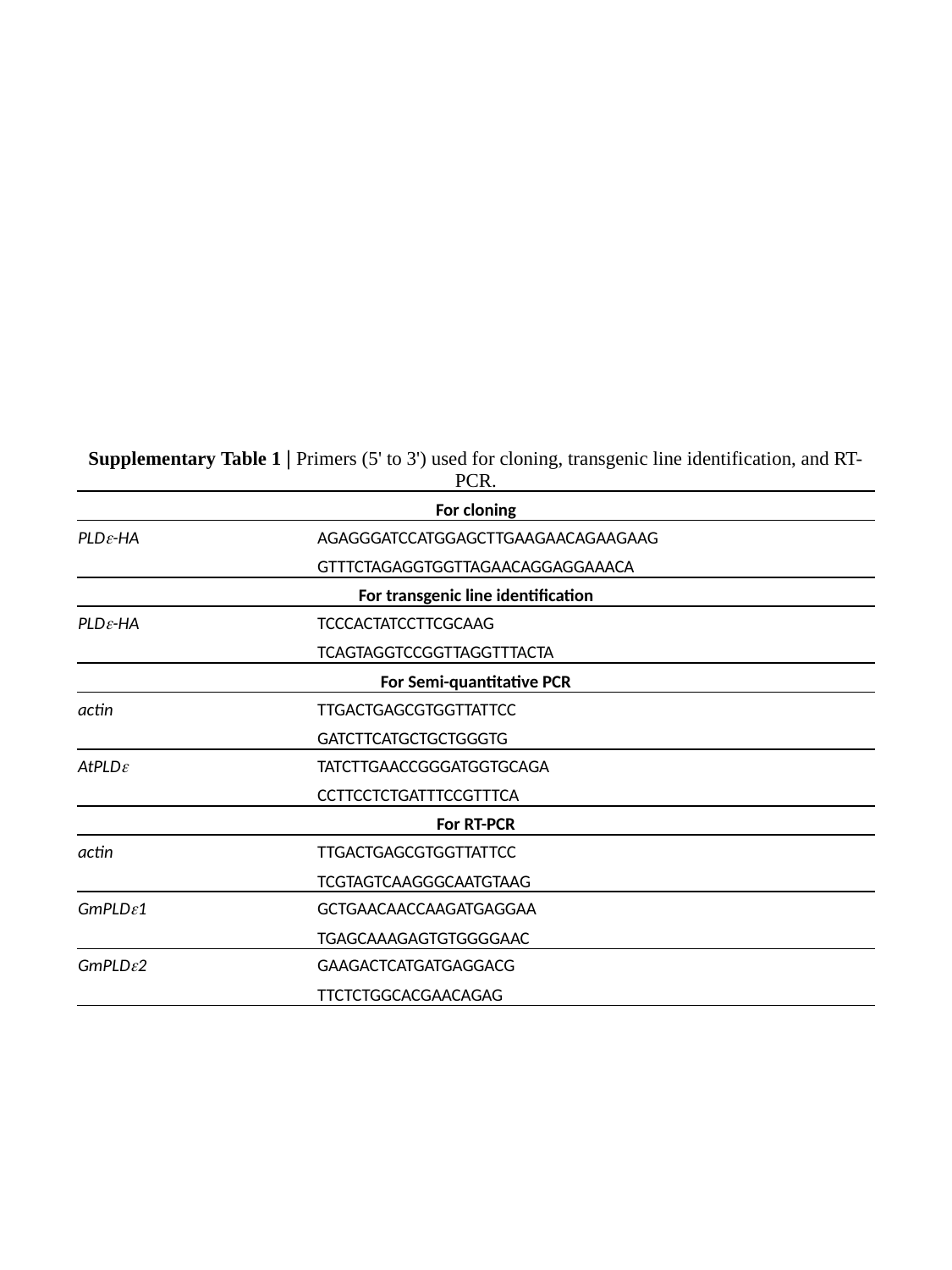

| Supplementary Table 1 | Primers (5' to 3') used for cloning, transgenic line identification, and RT-PCR. | |
| --- | --- |
| For cloning | |
| PLDe-HA | AGAGGGATCCATGGAGCTTGAAGAACAGAAGAAG |
| | GTTTCTAGAGGTGGTTAGAACAGGAGGAAACA |
| For transgenic line identification | |
| PLDe-HA | TCCCACTATCCTTCGCAAG |
| | TCAGTAGGTCCGGTTAGGTTTACTA |
| For Semi-quantitative PCR | |
| actin | TTGACTGAGCGTGGTTATTCC |
| | GATCTTCATGCTGCTGGGTG |
| AtPLDe | TATCTTGAACCGGGATGGTGCAGA |
| | CCTTCCTCTGATTTCCGTTTCA |
| For RT-PCR | |
| actin | TTGACTGAGCGTGGTTATTCC |
| | TCGTAGTCAAGGGCAATGTAAG |
| GmPLDe1 | GCTGAACAACCAAGATGAGGAA |
| | TGAGCAAAGAGTGTGGGGAAC |
| GmPLDe2 | GAAGACTCATGATGAGGACG |
| | TTCTCTGGCACGAACAGAG |
